# Impressions from a Field Ambulance

**Published:** 1915-01-16

**Authors:** 


### IMPRESSIONS FROM A FIELD AMBULANCE.

### Tyres, Equipment, and the Health of an Army.

BY AN OFFICER OF THE R.A.M.C. AT THE FRONT.

The number of topics connected with the work of the Royal Army Medical Corps in the present war, upon which discussion and comment are rife in the officers' messes of field ambulances and other medical units, is naturally very large. In selecting one or two at haphazard, it should be premised that they are not necessarily those of greatest importance, but simply such matters as happen to have recently obtruded themselves upon the writer in his actual experience.

### Ambulance Tyres.

The motor ambulance waggon now does much of the work which at the commencement of the campaign was perforce allotted to the horsed waggons of the field ambulances. This substitution is for the benefit of all concerned: of the wounded in transit, the officers and men of the ambulances, and of those in the fighting line amongst whom potential casualties may occur. Nevertheless, the horsed vehicle is still indispensable for many purposes; there are still functions of a field ambulance, that is to say, which cannot yet be carried out by motor transport and possibly never will be. The horsed waggon, therefore, is still a necessity, as far as this war is concerned at the least. That which is in use in the British Army has undergone several successive modifications since the South African War, which have greatly improved it in all sorts of respects. To discuss here the whole question of the construction of an ideal ambulance waggon is impossible; but there is one small point on which a note may be useful, and that is on the question of tyres.

The writer happened to travel to France on a troopship which carried two field ambulances, one of which had its waggon wheels equipped with (solid) rubber tyres, while the other had the older pattern of iron tyre. An officer of very considerable experience on board, speaking on this item of difference, expressed himself very strongly in favour of the old-fashioned tyre, on the ground that the rubber ones would not stand the heavy wear they would be sure to encounter. The time has come when it is possible to examine this contention in the light of actual fact. The solid rubber tyres have now had four months of incessant wear, on roads for the most part abominable, and in climatic conditions which have varied from torrid heat to some degrees of frost, and from long drought to heavy rain and snow. They have, moreover, in addition, undergone one railway journey in England and two in France, and the latter, in view of the dreadful state of the French rolling stock, with its barbarous side loading and its appalling *chevaux de frise* of tenpenny nails and other atrocities, is about as severe a test as any heavy vehicle can be put through. They have also travelled by sea a distance of nearly two hundred miles.

It is most satisfactory to be able to say that the tyres have stood the test magnificently. Not a single one has had to be replaced as yet in the particular ambulance with which the writer has worked. Not one (out of forty such tyres) is yet worn out, or anywhere near it; as far as can be judged they should last at least another four months, or even more, before renewal will be necessary. On one or two occasions, especially in connection with entrainments or embarkations, some sudden lateral strain has forced the solid tyre out of the slot on the wheel into which it is bedded, but on each occasion the special lever provided for this emergency has proved equal to the task of reducing the dislocation. In a word, the rubber tyre is triumphantly vindicated as far as its durability and suitability for active service conditions in a temperate climate are concerned.

As to its advantage from the point of view of the transport of the sick there are no two opinions. Not all the ingenuity of its designers has yet made the horse-drawn ambulance waggon an ideal vehicle for the carriage of sick or wounded men, though admittedly great mitigation of its discomforts has been achieved in the latest patterns. But the rubber-tyred waggon is many degrees better in this respect than the older type with metal-tyred wheels, so that, with its wearing capacity now proved, it may be pronounced definitely the more valuable by far of the two.

### Wheels, Saddles, Shoes.

Before passing on from this topic of wheels, it is worth while drawing attention to the fact that the establishment of a field ambulance, with its twenty-three vehicles and its seventy horses (roughly speaking) contains no provision for a farrier, a saddler, or a wheeler. This extraordinary omission means that if a horse casts a shoe, or a waggon-wheel comes loose, or a piece of harness wants mending, there is no one among the *personnel* who is instructed how to carry out these necessary functions. Anyone who has ever had control of wheeled transport of any sort will easily recognise the straits to which a field ambulance is inevitably reduced whenever any one of these common incidents of road travel is encountered. As a matter of fact, the field ambulance in such an event is constrained to entreat the favour and the assistance of the nearest unit which possesses artificers of this kind, usually in practice an ammunition column, a battery, or a supply column. Such assistance is cordially rendered whenever circumstances permit, but oftentimes circumstances do not permit, and the whole system of medical aid to the wounded is then and thereby imperilled. One has seen ambulance horses with their feet tied up in nosebags (the said nosebags being thereby entirely ruined) to enable them to draw a waggon, after casting a shoe, without undue risk of going dead lame, and harness mended with a piece of string is not at all an unfamiliar sight in a field ambulance. Whenever a lull in the field operations offers an opportunity, the prudent commander borrows the services for two or three days of a farrier, a saddler, and a wheeler, from the nearest unit which can spare them. The transport of the ambulance is thoroughly overhauled, and all makeshifts replaced by more adequate workmanship. This reliance upon happy chance has, as it happens, brought most of the field ambulances through more or less satisfactorily and fortunately; but it would unquestionably be far better to attach a farrier to every such unit, and, if possible, a saddler and wheeler as well.

### The Health of the Army.

The present campaign has differed radically and profoundly, so far, from the South African War, in that the casualties from the enemy's fire have been many times heavier than those from disease; whereas in South Africa disease accounted for more than twice as many victims as did warfare, if memory serves me rightly. This is, at first sight, all the more remarkable in that the climatic conditions in Flanders during the last two months have been far more unfavourable than those which prevailed in South Africa, at any rate on the high veld, where the greater part of our troops were resident. There has been, especially lately, an appreciable amount of rheumatism and of pulmonary complaints; but the marvel is, when the conditions are observed at first hand, that any of the troops in the trenches remain well at all. Of epidemic disease there has been extraordinarily little; and this is all the more remarkable in that the very rudiments of sanitation appear to be quite unknown—or at least unhonoured—by the authorities and populations of those parts of France and Belgium in which the military operations have been taking place.

One battalion, it is said by those likely to know, has had to be quarantined very strictly for some time owing to an outbreak of an infectious fever more usually associated with youth than with adult life. This outbreak, it is stated on good authority, was due to the soldiers taking up their quarters in a certain school which had been closed shortly before on account of that very disease amongst the children. The local authorities did not think it at all necessary to warn the British commander in any way about the epidemic, with the result already mentioned; and this happy-go-lucky attitude towards zymotic disease appears to be characteristic of the whole sanitary system (if system it can properly be called) in French and Belgian Flanders.

### Typhoid Fever.

Of that bugbear of armies, and especially of armies that are not on the march, typhoid fever, it is perhaps prudent not to say too much at present. It is common knowledge that there is typhoid fever among the Germans. This much we know for certain, since they left behind them cases of this disease in their deserted hospitals in various places, notably at Braisne, on the Aisne, and at other places in the north-western sphere of operations. At one town in Flanders they even left moribund typhoid patients dying untended in barns and similar places, possibly with the idea of disseminating typhoid among our incoming divisions. This much we can speak to positively as facts, and it naturally inclines us to believe the accounts received through other sources as to large epidemics in Lille and elsewhere.

### Sanitation.

It would not, then, be at all astonishing were an epidemic to break out in our Army, especially if the present German line is pushed back by an advance of our troops. It has also to be remembered that there is a fair amount of typhoid in the Belgian Army, and that it is more or less endemic in the unsanitary regions where our troops are housed. Strange to say, and most fortunately, there have been amazingly few cases among the British troops, and those have been scattered cases. Nothing even remotely approaching an epidemic has been recorded so far, and it is to be hoped this happy state of affairs will continue indefinitely. At least, every step that experts in sanitation can suggest is being taken to preserve the clean sheet, and it will not be for want of forethought if any considerable outbreak should occur. How far preventive inoculation accounts for this immunity is a statistical matter which we shall not be able to settle until after the war, but it is at least quite likely that this process has had a large share in keeping off the ravages of this insidious foe.

Dysentery, or at least an acute diarrhœa with slimy and sanguinolent evacuations and pyrexia, was accountable for a considerable number of non-effectives in the early autumn, probably because of the unusual heat and dryness of the season. Latterly very few such cases have been seen, and in this respect again the experience of the Boer war is quite reversed. So far, then, there is nothing but congratulation to pay our troops and their medical organisation, in so far as the incidence of disease, apart from wounds, is concerned. May this satisfactory state of things continue uninterrupted until the end of the war!

